# Shape-Adaptive Metastructures with Variable Bandgap Regions by 4D Printing

**DOI:** 10.3390/polym12030519

**Published:** 2020-03-01

**Authors:** Reza Noroozi, Mahdi Bodaghi, Hamid Jafari, Ali Zolfagharian, Mohammad Fotouhi

**Affiliations:** 1 Department of Engineering, School of Science and Technology, Nottingham Trent University, Nottingham NG11 8NS, UK; reza.noroozi@ut.ac.ir; 2 School of Mechanical Engineering, Faculty of Engineering, University of Tehran, Tehran 1417466191, Iran; hamid.jafari@ut.ac.ir; 3 School of Engineering, Deakin University, Geelong, VIC 3216, Australia; a.zolfagharian@deakin.edu.au; 4 School of Engineering, University of Glasgow, Glasgow G12 8QQ, UK; mohammad.fotouhi@glasgow.ac.uk

**Keywords:** 4D printing, metastructure, shape-memory polymers, wave propagation, finite element method, bandgap

## Abstract

This article shows how four-dimensional (4D) printing technology can engineer adaptive metastructures that exploit resonating self-bending elements to filter vibrational and acoustic noises and change filtering ranges. Fused deposition modeling (FDM) is implemented to fabricate temperature-responsive shape-memory polymer (SMP) elements with self-bending features. Experiments are conducted to reveal how the speed of the 4D printer head can affect functionally graded prestrain regime, shape recovery and self-bending characteristics of the active elements. A 3D constitutive model, along with an in-house finite element (FE) method, is developed to replicate the shape recovery and self-bending of SMP beams 4D-printed at different speeds. Furthermore, a simple approach of prestrain modeling is introduced into the commercial FE software package to simulate material tailoring and self-bending mechanism. The accuracy of the straightforward FE approach is validated against experimental observations and computational results from the in-house FE MATLAB-based code. Two periodic architected temperature-sensitive metastructures with adaptive dynamical characteristics are proposed to use bandgap engineering to forbid specific frequencies from propagating through the material. The developed computational tool is finally implemented to numerically examine how bandgap size and frequency range can be controlled and broadened. It is found out that the size and frequency range of the bandgaps are linked to changes in the geometry of self-bending elements printed at different speeds. This research is likely to advance the state-of-the-art 4D printing and unlock potentials in the design of functional metastructures for a broad range of applications in acoustic and structural engineering, including sound wave filters and waveguides.

## 1. Introduction

In order to survive in variable environments and keep their performance, natural materials have evolved to be active and adaptive, retaining functions across a range of stresses or strains or changing thermomechanical properties in response to external stimuli like light, temperature or moisture [1]. Several active materials have been developed to mimic the unique properties of natural materials and adaptive structures [2]. Among these active materials, shape-memory polymers (SMPs) have attracted much attention due to their lower density, higher recoverable strain of up to 400%, lower cost, simple shape programming procedure, and excellent controllability over the recovery temperature [3,4].

In the recent two decades, three-dimensional (3D) printing technology, also known as additive manufacturing (AM), has gained considerable attention as an advanced manufacturing technique that can create complex objects through depositing materials in a layer-by-layer manner [5,6,7,8,9]. With the introduction of active materials, 3D printing approaches have shown excellent potential for the fabrication of adaptive structures, namely four-dimensional (4D) printed structures, with the capability of reshaping their configuration and changing their properties over time [10,11,12]. For the first time, Tibbits [13] experimentally demonstrated how 4D-printed objects could transform over time and perform self-assemblies. While 3D printing methods can be used to fabricate static structures, 4D printing methods allow the fabrication of dynamically reconfigurable architectures with desired functionality and responsiveness. Considering a specific application, 4D-printed objects can be designed to respond to environmental stimuli and external triggers such as humidity, light, heat, and electric or magnetic fields [14,15,16]. For example, by 4D printing temperature-sensitive SMPs, Bodaghi et al. [17] introduced adaptive metamaterials with the capability of 1D/2D-to-2D/3D shape-shifting through self-folding and/or self-coiling. Wang et al. [18] introduced a novel 4D printing technology of composites with continuous embedded fibers for which the programmable deformation was caused by the difference in coefficient of thermal expansion between continuous fibers and flexible matrix. Zhang et al. [19] 4D-printed lightweight structures with self-folding/unfolding performances when exposed to a certain temperature. Also, Zhao et al. [20] 4D-printed SMPs by stereolithography of photopolymers. Fold-deploy test and shape-memory cycle measurements proved a high shape recovery rate, shape fixity, and recovery of the printed objects. Zolfagharian et al. [21] achieved controlled bending in a commercial prestrained SMP film by using different 4D-printed patterns and a number of layers. The photothermal stimulus was used to induce differential shrinking through the thickness of the actuator hinge. Recently, Wu et al. [10] designed a new 4D printable acrylate-based photosensitive resin prepared for digital light processing. They also investigated the influence of crosslinker concentration on the shape memory and mechanical properties.

Acoustic metamaterials are a new class of architected materials designed to control, direct, and manipulate waves through the material. Wave propagation in periodic metamaterials has been investigated broadly. Research works have revealed that these architected structures can be designed to gain desirable dispersion performance [22], lightweight structures [23], and excellent energy absorbers [24] by arranging unit cells in periodic repetition. The dispersion performance may present stop-band and pass-band frequency ranges as well. Phani et al. [25] introduced a computational procedure to find bandgaps by using Floquet–Bloch principles. Matlack et al. [26] fabricated metastructures by combining 3D printing polycarbonate lattices with steel cubes assembling. They showed that the value of bandgap could be varied by changing the lattice geometry and structural stiffness. By using deployable structures composed of beams and torsion springs, Nadda and Karami [27] showed that wave transmission characteristics could be tuned by modulating the fold angle. By combining a periodic lattice and locally-resonant inclusions with different temperature dependency, Nimmagadda and Matlack [28] proposed a metastructure that can tune the bandgaps under thermal stimuli.

The main objective of this research is to show how 4D printing technology can be used to engineer adaptive metastructures with the ability to control elastic wave propagation. Such structure is essential in vibration mitigation and acoustic attenuation. Inspired by thermomechanics of SMPs and the potential of fused deposition modeling (FDM) in 4D printing self-bending elements, adaptive functionally graded (FG) beams are fabricated. It is shown, experimentally and numerically, how 4D printing speed can control shape recovery and self-bending features of active elements. A 3D constitutive model, along with an in-house finite element (FE) code programmed in MATLAB software, is developed to replicate shape recovery of FG active elements 4D-printed at four different printing speeds. Afterward, a simple approach, along with the commercial FE software package of COMSOL Multiphysics, is introduced to simulate the material tailoring in the 4D printing stage and self-bending of active SMP elements. The accuracy and simplicity of the straightforward FE approach implemented in commercial FE software are assured via comparative studies with experimental data and numerical results from the in-house FE MATLAB-based code. Two periodic architected temperature-sensitive metastructures with adaptive dynamical characteristics are conceptually proposed. Their dynamic behaviors during heating–cooling processes are numerically studied in detail. Adaptive wave propagation (pass-band and stop-band), desired dynamic performance, and vibration manipulation are numerically demonstrated to be some of the unique characterizations of these metastructures. Computational studies reveal that, while the bandgaps in these metastructures are induced by the self-bending mechanism, their crucial feature is that the bandgap size and frequency range can be controlled and broadened through local resonances linked to changes in the structural geometry. The material/structural formulation, concepts, and results provided in this article are expected to be instrumental towards 4D printing adaptive metastructures for a broad range of applications, including structural vibration absorption, waveguiding, and noise mitigation.

## 2. Conceptual Design

### 2.1. Four-Dimensional Printing SMPs

In this section, by understanding the shape-memory effect (SME) and FDM technology, the FG 4D printing concept for designing adaptive structures is introduced. Temperature-sensitive SMPs are a class of smart materials that can recover their original shape from a temporary programmed shape by heating. In the programming step, the material, initially in a strain/stress-free state at a temperature lower than the transition temperature ($T<T_{g}$), is firstly heated up to $T_{h}$ that is higher than transition temperature ($T_{g}<T_{h}$). The material that is stable at the rubbery phase is then loaded and held fixed while being cooled down to *T_l_*, which is less than the transition temperature ($T_{l}<T_{g}$). By removing the constraints, an inelastic strain, so-called prestrain, remains in the material and forms an irregular shape. The material is in a free-stress state at this stage. In the shape recovery process, the SMP is heated to recover its original shape, which is known as free strain recovery, and finally is cooled back to the low temperature.

**Figure 1:**
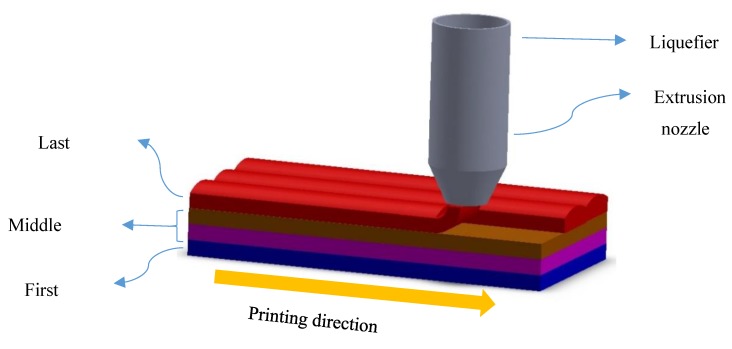
A schematic of the fused deposition modeling (FDM) method.

FDM technology, as a filament-based material-extrusion 3D printing method, applies a similar thermomechanical process on the material during the fabrication. Therefore, it may have the potential to fabricate 4D SMP architectures along with the shape programming. Figure 1 depicts a schematic of FDM technology. At first, the material is heated inside the liquefier up to $T_{\ln}$, which is higher than the transition temperature ($T_{g}$), and then forced out of the nozzle and deposited onto the platform by the 4D printer head moving at speed $S_{p}$. In this step, the material is stretched similar to the heating–loading process of the SMP programming step that induces the prestrain. Therefore, the printing speed may affect the prestrain value. It would be sensible that greater speed produces more significant mechanical loading, hence inducing greater prestrain. After deposition, the printed layer cools and solidifies in the same manner as the cooling step in the programming process. After 4D printing the layer, the platform moves downward, and the 4D printer head proceeds to deposit the following layer. The programming procedure is finalized by mechanical unloading through removing the printed object from the build tray.

The thermal/surface boundary condition between the 4D-printed layers may affect the through-the-thickness prestrain regime. For instance, while the first printing layer is deposited on the stiff and rough build tray, other layers above it are laid on the previously printed polymeric layers. Therefore, material and surface conditions may affect bonding and stretching conditions, reducing the first layer prestrain. The first layer is expected to show the lowest prestrain value. By printing the second layer, the first layer is partially reheated, and this extra heat may reduce the prestrain value. In other words, the first layer and layers above it, except the end layer, are always reheated, and their prestrain value is decreased. Since the last layer never gets any extra heat, it is expected to have the maximum prestrain. It may be concluded that the prestrain regime may have an increasing trend through the thickness upward from the lower to the upper layer. This additive manufacturing process can be called FG 4D printing as the material is programmed during the fabrication in the same manner as an FG material.

### 2.2. Material Behaviors

In this research, we used the polylactic acid (PLA) filament with a diameter of 1.75 mm and glass transition temperature of 65 °C. Objects were fabricated using a 3Dgence DOUBLE printer developed by 3Dgence. This is a low-cost desktop 3D printer that extrudes 1.75 mm filaments with a 0.4 mm nozzle. The 3Dgence Slicer software was used to produce G-code files from STL files and command and control parameters of liquefier temperature and printing speed. Beam-like elements were 4D-printed with dimensions of (30 × 1.6 × 1) mm for length, width, and thickness, respectively. Each printing layer was considered to have a 0.1 mm thickness. The print raster was assumed to be along the length direction. The temperature of the liquefier, build tray, and chamber were set 210, 24, and 24 °C, respectively. In all the 4D printings, unless otherwise stated, the printing speed was set to 5 mm/s in which the printer does not induce any prestrains in the materials.

**Figure 2:**
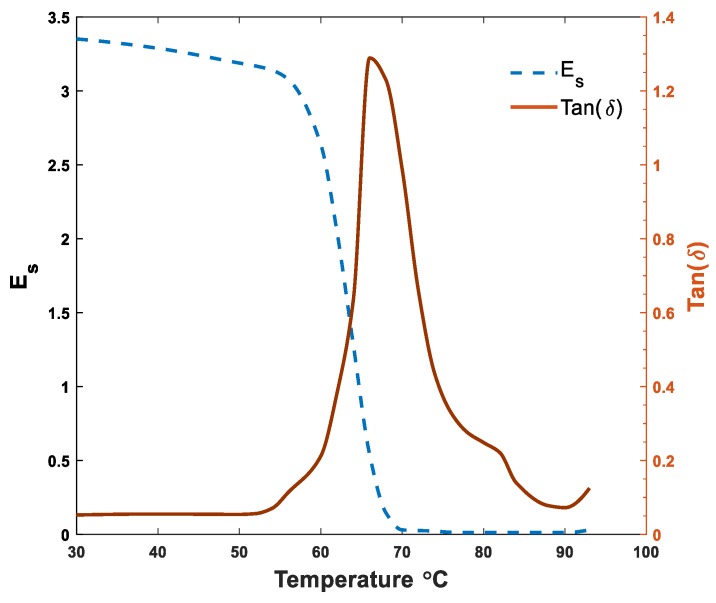
Dynamic-mechanical analyzer (DMA) test for the 3D-printed polylactic acid (PLA).

Dynamic-mechanical analyzer (DMA, Q800, TA Instruments, New Castle, DE, USA) was first implemented to determine the temperature-dependent mechanical properties of 3D-printed PLAs. DMA test was conducted in an axial tensile way with the frequency of force oscillation 1 Hz and heating rate 5 °C/min ranging from 30 to 93 °C. The applied dynamic stress was 1.5 times the static stress. Further, the thermomechanical behavior of the 4D-printed sample in terms of storage modulus, *E_s_*, and phase lag, tan(*δ*), is shown in Figure 2. It is seen that the storage modulus is reduced drastically as the material is heated to 60 °C. It is also observed that the phase lag graph peaks at 65 °C, which is assumed to be the glass transition temperature.

**Figure 3:**
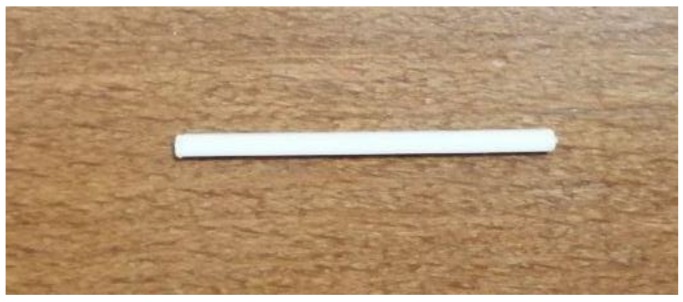
The beam configuration after 4D printing.

**Figure 4:**
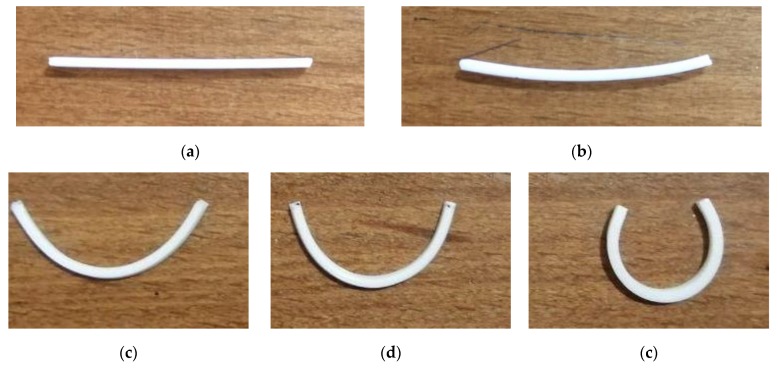
The configuration of the samples 4D-printed at different speeds of (**a**) 5, (**b**) 10, (**c**) 20, (**d**) 40, and (**e**) 70 mm/s after the heating–cooling process.

Five beam-like elements were 4D-printed at five different speeds (Sp = 5, 10, 20, 40, 70 mm/s). Figure 3 shows the beam configuration after 4D printing. After 4D printing, five samples were heated by dipping into the hot water with a prescribed temperature of 85 °C (20 °C higher than the transition temperature) and then were cooled down to the room temperature, 24 °C. Figure 4 depicts the sample configuration after the heating–cooling process. As can be seen, samples with a straight temporary shape may transform into curved beams. This means that the samples may already be programmed and prestained during the 4D printing process. Figure 4a shows that the sample printed at a low speed of 5 mm/s does not undergo any changes by heating. This implies that this printing speed is not enough to produce any prestrains. However, the configuration presented in Figure 4b indicates that increasing the printing speed to 10 mm/s can cause a slight curvature after heating. Thus, this speed can be considered as a transient speed. The configuration change could be due to an unbalanced FG prestrain regime induced through the thickness direction during the 4D printing process. When dipping the samples into hot water, the unbalanced prestrain, with an increasing trend through the thickness upward from the lower layer to the upper layer, was recovered, leading to a self-bending movement. The mismatch in free-strain recovery inducing curvatures enabled the overall configuration to change toward the top layer. It is worthwhile to mention that an increase in the 4D printing speed increased the bending angle and curvature. The faster the 4D printing, the greater the prestrain and consequently the deformation. Finally, experiments revealed that the FDM 4D printing technology has high potential in fabricating and programming adaptive objects with self-bending features.

## 3. Theoretical Modeling

The 4D-printed metastructures with adaptive wave propagation feature can be designed based on the theoretical understanding of the FG programming process and SME of 4D-printed SMP elements. This section is devoted to developing constitutive models and mathematical formulation to predict SME and wave propagation in 4D-printed SMPs.

### 3.1. SMP Model

In this division, a phenomenological constitutive model presented initially in [17,29] is reformulated to describe shape programming and recovery processes in the 4D-printed structures. Analytical straightforward closed-form solutions are also derived.

The SMPs consist of two phases, so-called glassy and rubbery phases, which are stable at temperatures above and below the transition temperature, respectively. As SMPs show a mixture of glassy and rubbery phases, volume fractions of the rubbery and glassy phases describe the state of SMPs. The following scalar variables characterize them:

(1) $$\eta_{g}=\frac{V_{g}}{V}\;\eta_{r}=\frac{V_{r}}{V}$$

where $V_{g}$ and $V_{r}$ indicate the volume of the glassy and rubbery phases, respectively. The subscripts ‘*g*’ and ‘*r*’ stand for glassy and rubbery phases, respectively, here and henceforth. The change between these phases is considered to be only a function of temperature as a generally well-known assumption. It implies that $\eta_{g}$ and $\eta_{r}$ are just functions of the temperature. Because in every moment the summation of these parameters must satisfy the unity ($\eta_{g}+\eta_{r}=1$), the volume fraction of the rubbery phase can be expressed in terms of the glassy one as:

(2) $$\eta_{r}(T)=1-\eta_{g}(T)$$

Considering experimental results from the DMA test, $\eta_{g}$ can explicitly be interpolated by a hyperbolic function as [17,29]:

(3) $$\eta_{g}=\frac{\tanh(a_{1}T_{g}-a_{2}T)-\tanh(a_{1}T_{g}-a_{2}T_{h})}{\tanh(a_{1}T_{g}-a_{2}T_{h})-\tanh(a_{1}T_{g}-a_{2}T_{l})}$$

in which $a_{1}\mathrm{and}a_{2}$ are determined by adjusting the DMA curve.

The glassy and rubbery phases in SMPs are assumed to be linked in series, i.e., $\sigma =\sigma_{g}=\sigma_{r}$, where σ denotes the stress. Considering the fact that the 4D-printed objects may experience small strains and moderately large rotations, a small strain regime is assumed. Consequently, an additive strain decomposition is adopted as:

(4) $$\varepsilon =\eta_{g}\varepsilon_{g}+(1-\eta_{g})\varepsilon_{r}+\varepsilon_{in}+\varepsilon_{th}$$

where ε denotes the total strain while $\varepsilon_{g}$ and $\varepsilon_{r}$ designate the strains of the glassy and rubbery phases, respectively. Also, $\varepsilon_{in}$ is the inelastic strain due to the SMP phase transformation, while $\varepsilon_{th}$ indicates the thermal strain that can be expressed as $\varepsilon_{th}=\int_{T_{0}}^{T}\left(\alpha_{r}+(\alpha_{g}-\alpha_{r})\;\eta_{g}(T)\right)\;dT$ in which $\alpha_{r}\;\mathrm{and}\;\alpha_{g}$ denote thermal expansion in rubbery and glassy phases, respectively, and $T_{0}$ is the reference temperature.

Next, $\varepsilon_{in}$, associated with the glassy–rubbery phase transformation mechanism, will be formulated. During the cooling step, the rubbery phase changes into the glassy one, and its strain is stored in the SMP material. The strain storage is assumed to be proportional to $\eta_{g}$ based on the rubbery phase strain. In the heating step, the stored strain is assumed to be released gradually, proportional to $\eta_{g}$ concerning the preceding glassy phase and strain storage. Therefore, $\varepsilon_{in}$ can mathematically be formulated in a rating format as:

(5) $${\dot{\varepsilon }}_{in}=\left\{ \begin{array}{l} {\dot{\eta }}_{g}\;\varepsilon_{r}\;\dot{T}<0\\ \frac{{\dot{\eta }}_{g}}{\eta_{g}}\;\varepsilon_{in}\;\dot{T}>0 \end{array}\right.$$

By considering ε and T as external control variables and $\varepsilon_{g},\varepsilon_{r},\varepsilon_{in}$, and $\eta_{g}$ as internal variables, introducing Helmholtz free energy density functions, implementing the second law of thermodynamics in the sense of the Clausius–Duhem inequality, and following a standard argument [30], the stress state can be obtained as:

(6) $$\sigma =\sigma_{g}=\sigma_{r}$$

This equation is consistent with the taken assumption that the stress in each phase is equal in the series model. The glassy- and rubbery-phase stresses are also derived as:

(7) $$\sigma_{g}=C_{g}\varepsilon_{g}\;,\;\sigma_{r}=C_{r}\varepsilon_{r}$$

in which *C* signifies the elasticity stiffness matrix defined as:

(8) $$C=\frac{E}{\left(1+\nu )(1-2\nu \right)}\;\left[\begin{array}{cccccc} 1-\nu & \nu & \nu & 0 & 0 & 0\\ \nu & 1-\nu & \nu & 0 & 0 & 0\\ \nu & \nu & 1-\nu & 0 & 0 & 0\\ 0 & 0 & 0 & \frac{\left(1-2\nu \right)}{2} & 0 & 0\\ 0 & 0 & 0 & 0 & \frac{\left(1-2\nu \right)}{2} & 0\\ 0 & 0 & 0 & 0 & 0 & \frac{\left(1-2\nu \right)}{2} \end{array}\right]$$

where *E* and *v* denote Young’s modulus and Poisson’s ratio, respectively. The stress–strain relationship can be derived by substituting Equation (7) into Equation (4) as:

(9) $$\sigma ={\left(S_{r}+\eta_{g}(S_{g}-S_{r})\right)}^{-1}(\varepsilon -\varepsilon_{in}-\varepsilon_{th})$$

where S indicates the compliance matrix defined as $C^{-1}$.

From a numerical viewpoint, the nonlinear SMP behaviors can be treated in an explicit time-discrete stress/strain-temperature-driven framework. The time domain [0,t] is discretized into increments, and the evolution equation is solved over the local band $\left[t^{n},t^{n+1}\right]$. The superscript n+1 shows the current step, while the superscript n denotes the previous step. The inelastic strain can be computed by applying the linearized implicit backward-Euler integration method to the flow rule (5) as:

(10) $$\varepsilon_{in}^{n+1}=\left\{ \begin{array}{l} \varepsilon_{in}^{n}+\Delta \eta_{g}^{n+1}\;\varepsilon_{r}^{n+1}\;\dot{T}<0\\ \varepsilon_{in}^{n}+\frac{\Delta \eta_{g}^{n+1}}{\eta_{g}^{n+1}}\;\varepsilon_{in}^{n+1}\;\dot{T}>0 \end{array}\right.$$

where

(11) $$\Delta \eta_{g}^{n+1}=\eta_{g}^{n+1}-\eta_{g}^{n}$$

By using Equations (7) and (9) to substitute the rubbery strain and the stress, and performing some mathematical simplifications, $\varepsilon_{in}$ defined in (10) can be updated for cooling and heating steps in stress and strain control ways as:

**Cooling:**

(12) $$\mathrm{stress}\;\mathrm{control}\;\mathrm{mode}\to \varepsilon_{in}^{n+1}=\varepsilon_{in}^{n}+\Delta \eta_{g}^{n+1}S_{r}^{n+1}\sigma^{n+1}$$

(13) $$\mathrm{strain}\;\mathrm{control}\;\mathrm{mode}\to \varepsilon_{in}^{n+1}={\left(I+\Delta \eta_{g}^{n+1}S_{r}^{n+1}C_{e}^{n+1}\right)}^{-1}\;(\varepsilon_{in}^{n}+\Delta \eta_{g}^{n+1}\;S_{r}^{n+1}C_{e}^{n+1}\;(\varepsilon^{n+1}-\varepsilon_{th}^{n+1}))\;$$

**Heating:**

(14) $$\varepsilon_{in}^{n+1}=\frac{\eta_{g}^{n+1}}{\eta_{g}^{n}}\varepsilon_{in}^{n}$$

Finally, by considering updated inelastic strain, the stress–strain constitutive Equation (9) can be discretized and unified for heating and cooling processes as:

(15) $$\sigma^{n+1}=C_{D}^{n+1}(\varepsilon^{n+1}-\varsigma \varepsilon_{in}^{n+1}-\varepsilon_{th}^{n+1})$$

where the so-called unified stiffness matrix $C_{D}$ and parameter ς for cooling and heating processes are defined as:

(16) $$\left\{ \begin{array}{l} C_{D}^{n+1}={\left(I+\Delta \eta_{g}^{n+1}S_{r}^{n+1}C_{e}^{n+1}\right)}^{-1}\;C_{e}^{n+1},\;\varsigma =1\;\dot{T}<0\\ C_{D}^{n+1}=C_{e},\;\varsigma =\frac{\eta_{g}^{n+1}}{\eta_{g}^{n}}\;\dot{T}>0 \end{array}\right.$$

### 3.2. Wave Propagation Model

**Figure 5:**
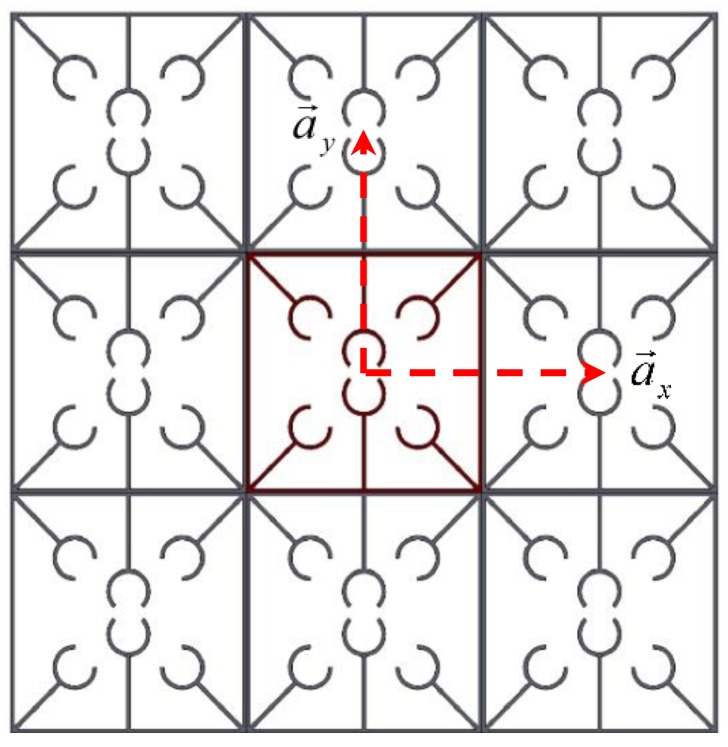
Direct vectors in a periodic model.

The wave propagation analysis of the architected periodic structures can be carried out by using the Bloch’s theorem for local resonance. Based on this theory, the displacement of each node in a chosen unit cell in the region of a periodic structure depends only on the displacement field of the equal node in the reference unit cell (${\vec{U}}_{\mathrm{Ref}}\left(\vec{r}\right)$). The formulation of this theorem is implemented to solve the equations of motion in the periodic boundary conditions. This concept is stated as [31]:

(17) $$\vec{U}\left(\vec{r}+\vec{R},t\right)={\vec{U}}_{\mathrm{Ref}}\left(\vec{r}\right)\exp\left(i\left[\vec{\kappa }.(\vec{r}+\vec{R})-\omega t\right]\right)$$

where $\vec{r}$ and $\vec{R}$ are position and lattice vectors, respectively. Also, the Bloch wave vector $\vec{\kappa }$ in the 2D periodicity is considered as $\vec{\kappa }=\left(\kappa_{x},\kappa_{y}\right)$, where $\kappa_{x}$ and $\kappa_{y}$ denote the phase constants which are measures of the phase variations over one unit cell in two directions of the periodicity (X–Y). Also, ω and t refer to frequency and time, respectively. As illustrated in Figure 5, the periodicity in two directions is defined by the direct vectors ${\vec{a}}_{x}$ and ${\vec{a}}_{y}$. Hence, the lattice vector is described as:

(18) $$\vec{R}=n_{x}{\vec{a}}_{x}+n_{y}{\vec{a}}_{y}\;,\;n_{x},n_{y}=0,\pm 1,\pm 2,\cdots$$

**Figure 6:**
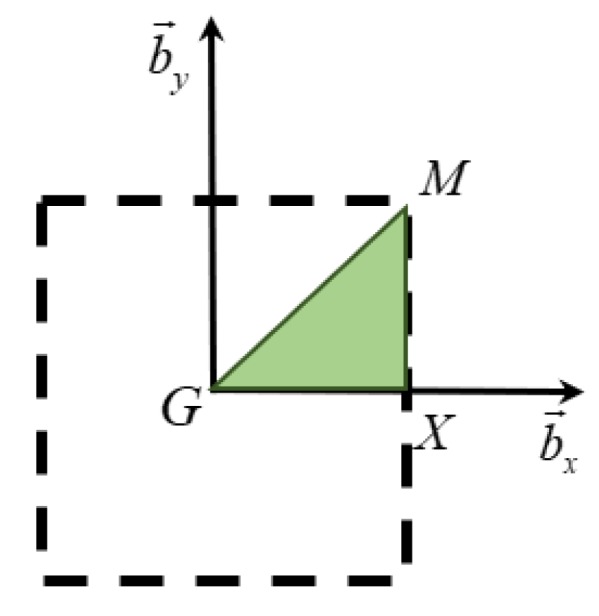
First Brillouin zone (FBZ, dashed square) and irreducible Brillouin zone (IBZ, colored triangle).

Since the Bloch wave vector varies in the Brillouin zone, the procedures to calculate reciprocal vectors and the first Brillouin zone are explained. Equation (19) formulates the reciprocal vectors ${\vec{b}}_{x}$ and ${\vec{b}}_{y}$ as [32]:

(19) $$\{\begin{array}{c} {\vec{b}}_{x}=\frac{2\pi }{\left|{\vec{a}}_{x}\times {\vec{a}}_{y}\right|}\left[\begin{array}{c} a_{yY}\\ -a_{yX} \end{array}\right]\\ {\vec{b}}_{y}=\frac{2\pi }{\left|{\vec{a}}_{x}\times {\vec{a}}_{y}\right|}\left[\begin{array}{c} -a_{xY}\\ a_{yX} \end{array}\right] \end{array}$$

where $\left(a_{xX},a_{xY}\right)$ and $\left(a_{yX},a_{yY}\right)$ are components of direct vectors ${\vec{a}}_{x}$ and ${\vec{a}}_{y}$ along the X- and Y-directions, respectively. The first Brillouin zone (FBZ) and irreducible Brillouin zone (IBZ) are depicted in Figure 6. The IBZ is the smallest part of the FBZ representing all the high-symmetry points in which the wave propagation is analyzed [33].

In this research, numerical simulations have been produced by employing an FE software. Each unit cell is discretized into finite elements. The dispersion relation is an eigenvalue problem that is solved By COMSOL Multiphysics 4.3. The problem has three unknown parameters ($\kappa_{x}$, $\kappa_{y}$, and ω) in which the wave vectors are examined in the IBZ (G-X-M-G), and the problem is determined to calculate the eigenfrequencies. Convergence studies of the mesh size have also been performed to achieve the converged results accurately to three significant digits.

### 3.3. FE Solution

#### 3.3.1. In-House FE Code

In order to replicate thermomechanical behaviors of FG 4D-printed elements, a Ritz-based FE solution is developed in MATLAB software. A 3D 20-node quadratic serendipity hexahedron element is considered in this problem. It has 20 nodes so that 8 corner nodes are augmented with 12 side nodes located at the side center. The element also has 3 degrees of freedom per node ($u_{i}$, i = 1, 2, 3). More details on the FE solution and numerical programming can be found in [17].

#### 3.3.2. COMSOL Multiphysics FE Modeling

In this section, thermomechanical behaviors of 4D-printed samples are analyzed by implementing a simple method in COMSOL Multiphysics software. For this purpose, by using the DMA test data, the temperature-dependent Young’s modulus is implemented in the COMSOL Multiphysics. Table 1 shows the dependency of Young’s modulus on the temperature.

**Figure 7:**
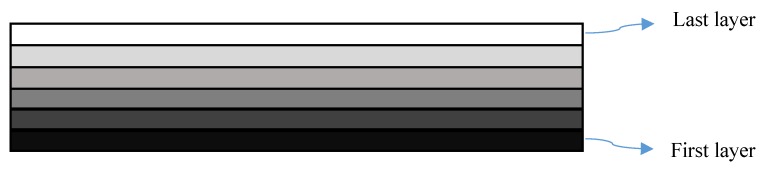
Discretized 4D-printed sample.

As described already, during 4D printing, a prestrain that varies through the thickness is induced in the object producing an FG structure. For modeling 4D-printed structures in COMSOL Multiphysics, the object can be divided into multiple sections with variable thermal expansion. In this study, the 4D-printed beam-like structures are divided into six sections. Figure 7 illustrates a discretized form of a printed beam with different thermal expansion coefficients.

The thermal expansion of each layer is chosen to replicate a configuration similar to the experiments as depicted in Figure 4 for a specific printing speed. Table 2 indicates the thermal expansion coefficient assumed for each layer.

A relationship between thermal expansion coefficients and printing speed can be formulated as:

(20) $$\alpha_{i}=C_{1}\;S^{3}{}_{P}+C_{2}\;S^{2}{}_{P}+C_{3}\;S_{P}+C_{4}\;10\leq S_{P}\leq 70$$

where C1, C2, and C3 are constants defined for each layer in Table 3.

**Figure 8:**
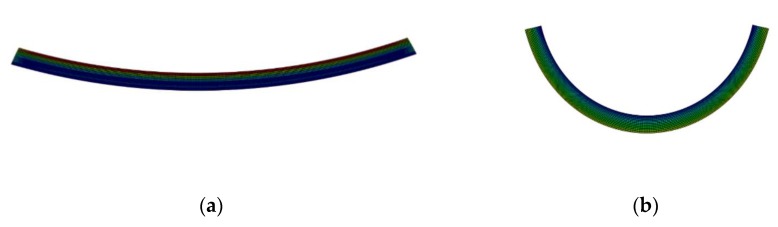

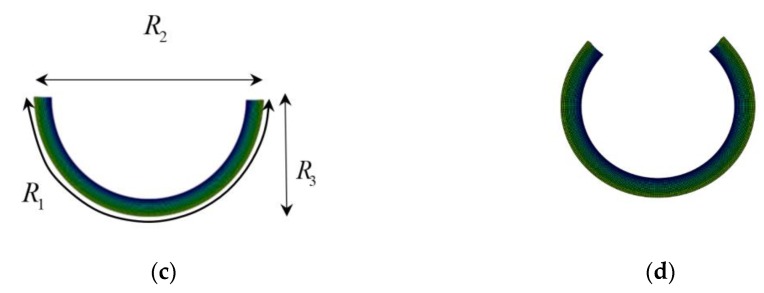
Finite element (FE) COMSOL Multiphysics simulation of the samples 4D-printed with different speeds of (**a**) 10, (**b**) 20, (**c**) 40, and (**d**) 70 mm/s after the heating–cooling process.

Figure 8 shows the deformed configuration obtained from the FE COMSOL Multiphysics simulation of self-bending 4D-printed beams after the heating–cooling process. In order to characterize the configuration of the printed samples after the heating–cooling process, three geometric parameters are considered. For this purpose, we use parameters $R_{1},R_{2}$, and $R_{3}$ which describe the outer length, opening, and depth of mid surface, respectively, as shown in Figure 8c. In order to determine the accuracy and efficiency of the simple method implemented in COMSOL Multiphysics, the geometric features obtained from the experiments, FE COMSOL Multiphysics, and in-house FE code are compared in Table 4. It can be concluded that the simulation results of FE COMSOL Multiphysics are in good agreement with those measured from experiments and calculated by the in-house FE solution. This way, the reliability and accuracy of setting variable thermal expansion coefficients in the COMSOL Multiphysics in replicating the self-bending feature observed in the 4D-printed samples is validated.

### 3.4. Periodic Structural Design

**Figure 9:**
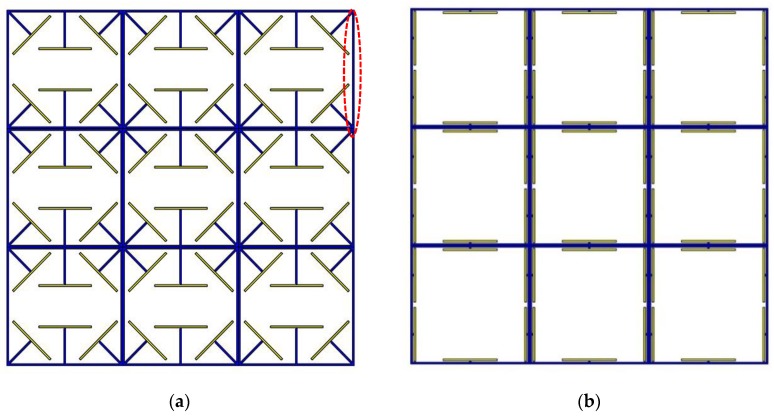
Periodic metastructures with active and passive components: (**a**) diagonal structure; (**b**) parallel structure (the red dashed oval shows the fixed-fixed beam used for the frequency normalization).

In this section, two periodic architected metastructures with adaptive dynamical characteristics are conceptually proposed. These metastructures are made of passive mainframes printed at a low speed so that no prestrain is induce, and active beam-like members printed at high speed with induced prestrains and self-folding features. They have the potential to be 4D-printed by setting different printing speeds for two nozzles. The PLA material as characterized in Section 2.2 and Section 3.3 are considered for 4D printing. The passive main frame of the metastructure is printed at a low speed such as 5 mm/s, while active elements with self-bending features are fabricated with three different 4D printing speeds. Different arrangements have been designed, and their dynamic performance has been examined numerically. Two metastructures have shown a high dynamic performance that will further be examined numerically in the next section. The first adaptive structure consists of active elements connected in parallel and diagonal to the frame, while the second adaptive structure consists of active elements connected in parallel with the frame. For convenience, they are called diagonal and parallel metastructures, respectively. Figure 9 shows the designed structures in which yellow and blue colors signify active and passive elements, respectively.

## 4. Results and Discussion

Periodic active structures are associated with granting propagating and bandgap ranges. In the propagating frequency ranges, the elastic wave propagation is done in all directions, but the bandgap represents a frequency range where the elastic wave propagation is stopped. In this section, COMSOL-based numerical results are presented, revealing how to design adaptive periodic structures with the ability to optimize the dynamical functionality without embedding any additional resonating components. Since it is difficult to actuate all mode shapes in experimental studies, experimental works have been considered in-plane or out-of-plane mode shapes only. However, in the present numerical study, a 3D dynamic case is investigated by FE COMSOL Multiphysics, and all modes of vibrations (i.e., bending, torsion, and elongation) can be measured without any limitation.

**Figure 10:**
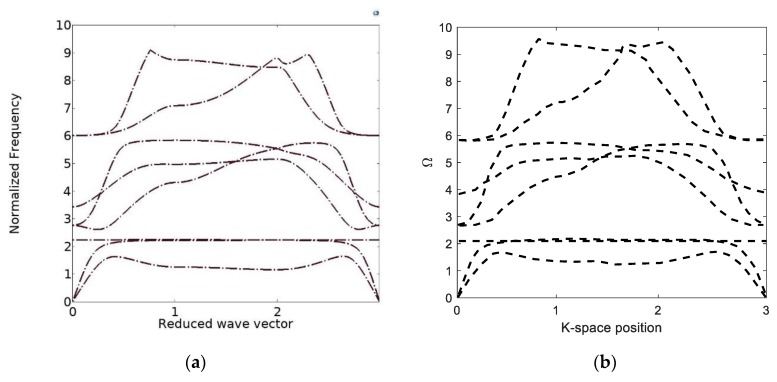
Verification of the band structure of the triangular topology: (**a**) the current study; (**b**) Ref. [25].

In order to verify the FE simulation, the bandgap of a triangular structure is simulated, as shown in Figure 10, and compared with the results of [25]. It is seen that there is an excellent agreement between the dispersion curves from the FE simulation and [25].

**Figure 11:**
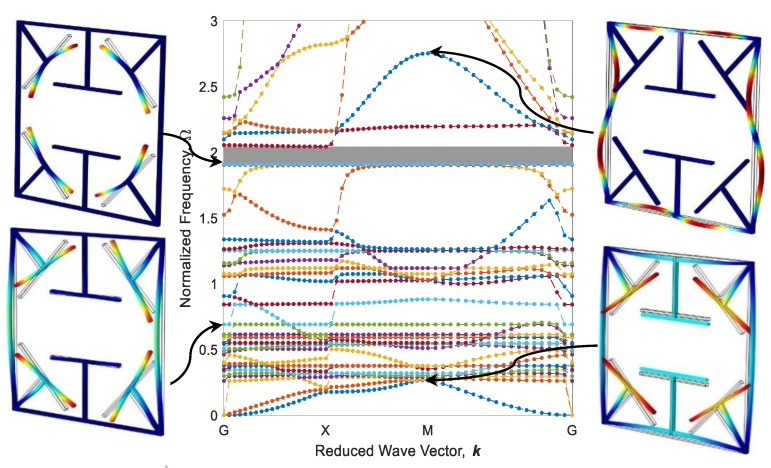
Band structure and mode shapes of the diagonal metastructure.

Eigenfrequencies of adaptive periodic structures, as shown in Figure 9, are computed by imposing periodic boundary conditions in different elastic wave vectors which are estimated based on IBZ detailed in the previous section. These eigenfrequencies are finally normalized against $\Omega =\omega \;/\omega_{0}$, in which $\omega_{0}=22.4\sqrt{EI/\left(mL_{0}^{4}\right)}$ is the first natural frequency of a fixed-fixed single beam, as shown in Figure 9a. The dispersion diagram and some out-of-plane mode shapes of the diagonal metastructure are illustrated in Figure 11, whereas the mode shapes are depicted in high symmetry points (G, X, or M). As can be seen, there is a wide bandgap (gray square) in the range of Ω=1.902−2.043 which is 4.68% of the frequency ranging between 0 and 3. As shown in Figure 11, the cause of bandgap in this range is the resonance of active parts. Further, there is a flat eigenfrequency before the bandgap which helps to assure its local resonance nature.

**Figure 12:**
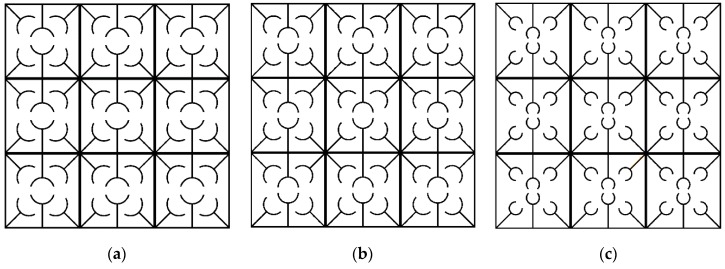
The configuration of adaptive periodic diagonal metastructure after heating–cooling process for three different printing speeds: (**a**) 20, (**b**) 40, and (**c**) 70 mm/s.

**Figure 13:**
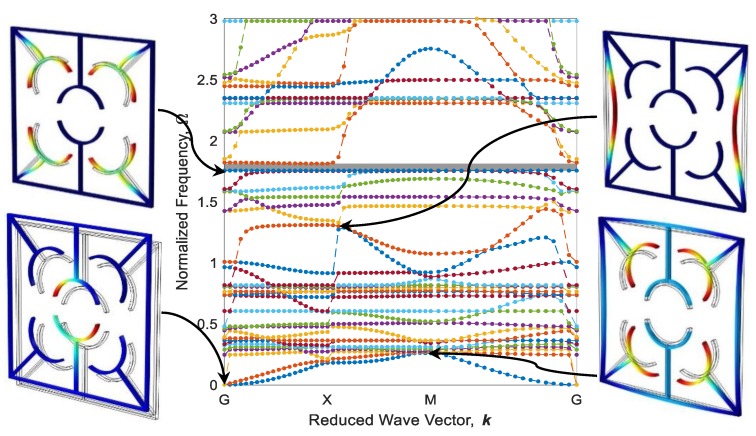
Band structure and mode shapes of the diagonal structure with active elements printed at 20 mm/s after the heating–cooling process.

**Figure 14:**
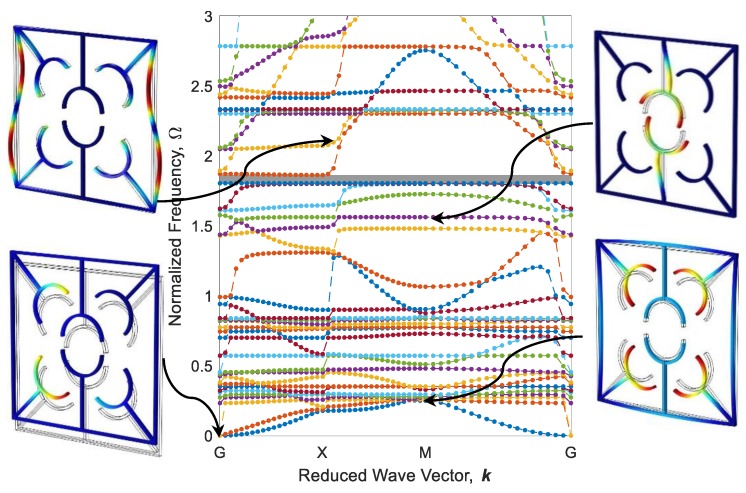
The counterpart of Figure 12 for 40 mm/s.

**Figure 15:**
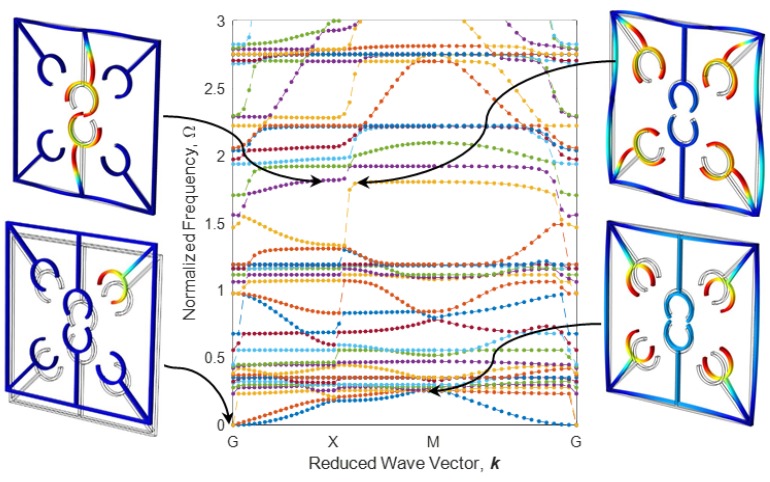
The counterpart of Figure 12 for 70 mm/s.

The configuration of the diagonal structure after the heating–cooling process for three printing speeds of active elements is illustrated in Figure 12. As it is expected, the elements 4D-printed faster produce more curvature after thermal activation. It is worth mentioning that the heating could also be another variable to control the curvature. i.e., by partially heating the metastructure with active elements printed at 70 mm/s speed, configurations become like those of thoroughly heated structures with active elements printed at 20 and 40 mm/s. Dispersion curves of diagonal structure with active elements of different 4D printing speeds after the heating–cooling process are depicted in Figure 13, Figure 14 and Figure 15. Comparing the results presented in Figure 11 and Figure 13 reveal that the bandgap area is adapted from Ω=1.902−2.043 to Ω=1.751−1.812, and the expanse of the bandgap area decreases from 4.68% to 2.01% as well. This phenomenon shows the significant effect of the self-bending feature on the locally resonant filtration. As can be seen, the functional range changes by tuning the 4D printing speed of active elements.

Figure 13 and Figure 14 reveal that increasing the 4D printing speed from 20 to 40 mm/s does not affect the bandgap range much, changes the stop-band area from 2.01% to 2.07%, and moves the range of bandgap to Ω=1.801−1.863. However, Figure 12c and Figure 15 show an interesting point: after heating–cooling of the diagonal metastructure with the printing speed of 70 mm/s, the whole stop-band area is vanished, and this model allows all the frequencies in the range of 0–3 times of reference frequency to pass. Also, the mode shapes of the structure in some of the high symmetry points and before the bandgap area are depicted in Figure 13. The results presented in Figure 11, Figure 12, Figure 13, Figure 14 and Figure 15 imply that varying 4D printing speed or heating temperature in the diagonal structure changes the dispersion behaviors significantly and can be manipulated to find an appropriate locally resonant vibration filter. The phenomenon of bandgap switch caused by changing the natural frequency of the active part in the periodic structure is diminished by local resonance changing frequency in different printing speeds.

**Figure 16:**
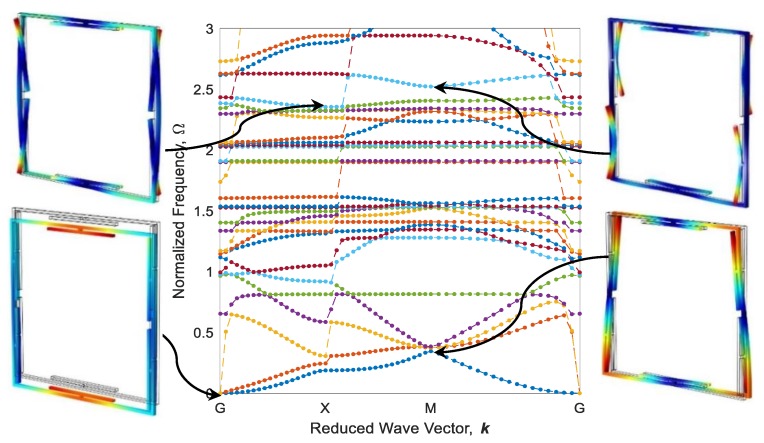
Band structure and mode shapes of the parallel metastructure.

**Figure 17:**
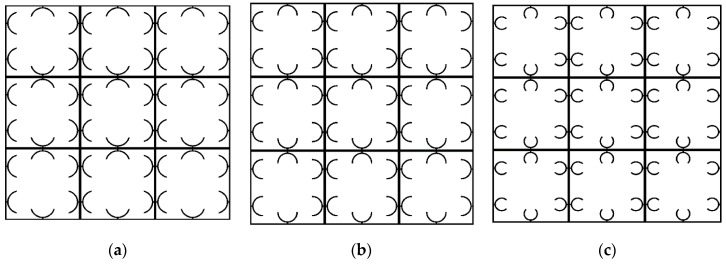
The configuration of adaptive periodic parallel metastructure after heating–cooling process for three different printing speeds: (**a**) 20, (**b**) 40, and (**c**) 70 mm/s.

**Figure 18:**
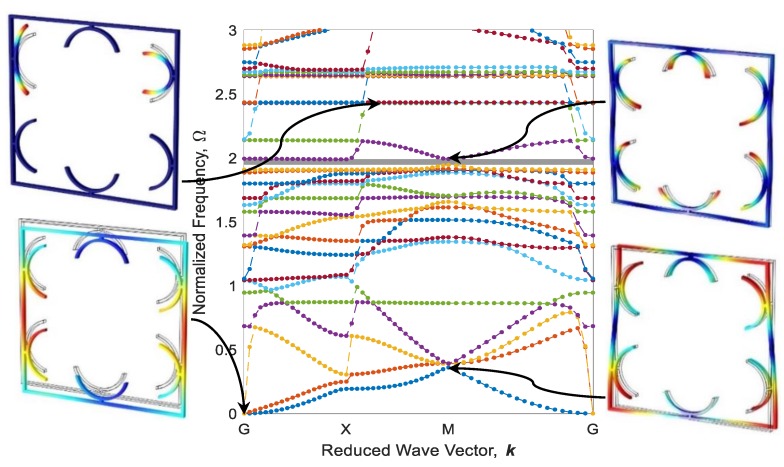
Band structure and mode shapes of the parallel metastructure with active elements printed at 20 mm/s after the heating–cooling process.

**Figure 19:**
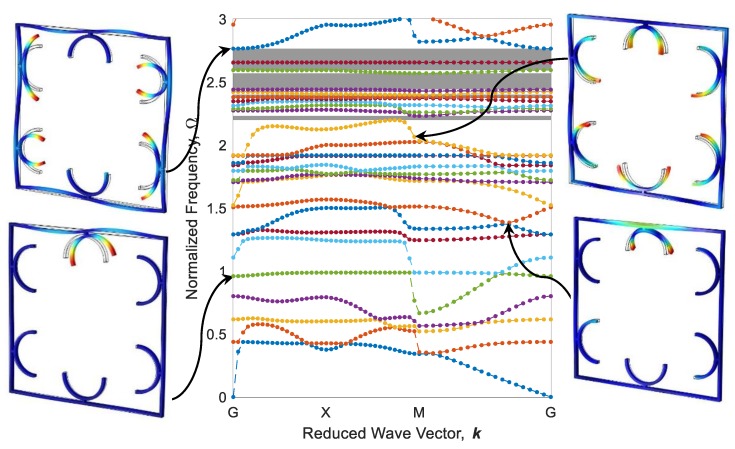
The counterpart of Figure 17 for 40 mm/s.

**Figure 20:**
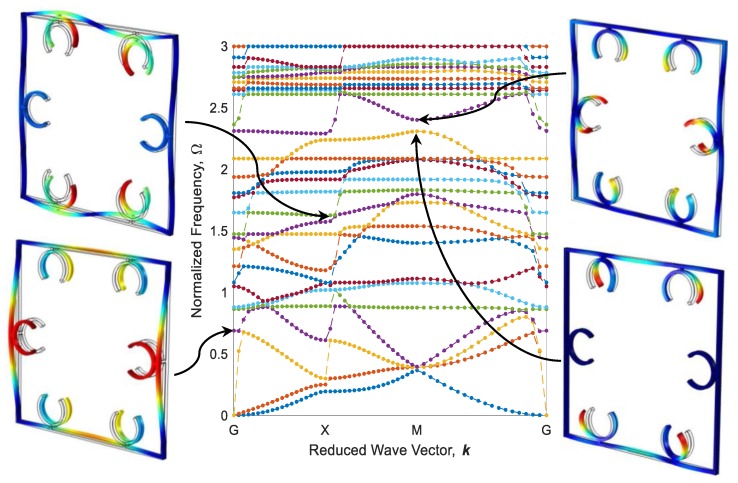
The counterpart of Figure 17 for 70 mm/s.

The parallel metastructure, as shown in Figure 9b displays different dispersion behaviors than the diagonal metastructure (Figure 9a). The dispersion curve for this model is illustrated in Figure 16, where there is no bandgap area, meaning that this structure allows all elastic waves in the frequency range of 0 to 3 to pass. Like diagonal metastructures, the active elements of the parallel structure are also manufactured with three different printing speeds. The configuration of parallel metastructures after the heating–cooling process is depicted in Figure 17, where parts a–c represent the self-bending metastructures 4D-printed at the speeds of 20, 40, and 70 mm/s, respectively. Furthermore, the band structure and mode shapes of parallel structures with self-bending elements of different printing speeds 20, 40, and 70 mm/s after the heating–cooling process are depicted in Figure 18, Figure 19 and Figure 20, respectively.

As it can be seen in Figure 16 and Figure 18, Figure 19 and Figure 20, the dynamic behaviors of these metastructures are remarkable such that the bandgap area has an increasing–decreasing trend as the structure is heated, revealing self-bending features. Figure 18 shows that the actuated metastructure with self-bending elements 4D-printed at 20 mm/s has a narrow bandgap area in the range of Ω=1.945−1.989 with the amount of 1.48%. However, by using active elements with a higher printing speed of 40 mm/s, the dynamic behaviors change. It is found that the amount of bandgap area increases to 12.32%, and the system exhibits stop-bands in multiple frequencies (Figure 19). These ranges are read as Ω=2.172−2.231, Ω=2.371−2.505, Ω=2.421−2.430, Ω=2.441−2.569, and Ω=2.594−2.765. This implies that this design has a better performance than the others. It can be concluded that this type of 4D-printed architected metastructure has excellent potential in adapting its locally resonant filters from 0 to a significant value such as 12.32%. These bandgaps are generated by Bragg scattering within the medium. In this type of adaptive periodic structure, there is no locally resonant bandgap and the bandgaps are the Bragg type.

Finally, the numerical results presented in Figure 20 reveal that by using active elements with a 4D printing speed of 70 mm/s, the bandgap area vanishes. This means that this metastructure, such as the structure before heating–cooling, as shown in Figure 16, propagates all the locally resonant vibration in all directions.

## 5. Conclusions

This article was aimed at 4D printing adaptive metastructures with locally resonated and Bragg-type stop-bands. The FDM 4D printing technology was implemented to program shape-memory elements during the layer-by-layer deposition process in a functionally graded manner. Experiments were conducted to explore 1D-to-2D self-bending features characterized in terms of 4D printer head speed. Boundary value problems were solved to explain thermomechanical mechanisms behind inducing prestrain during 4D printing and shape recovery after thermal activation. In this respect, a straightforward approach was introduced and implemented into the commercial FE software package of COMSOL Multiphysics, which is much simpler than writing a user-defined material model (UMAT) subroutine or an in-house FE code. The 4D-printed elements were simulated as functionally graded materials whose thermal expansion changed through the thickness direction. The excellent accuracy of the proposed technique was checked via a comparative study with experiments and computational results from the developed in-house FE MATLAB-based solution. Two periodic architected temperature-sensitive metastructures with adaptive dynamical characteristics were conceptually proposed. The COMSOL-based computational tool was then applied to dynamically analyze periodic metastructures with self-bending active elements 4D-printed at different printing speeds. It was found that the metastructures have the capability of controlling elastic wave propagation by forming bandgaps or frequency ranges where the wave cannot propagate. It was observed that the bandgap size and frequency range could be controlled and broadened through local resonances by changing 4D printing speed and thermal excitation. Due to the absence of a similar concept and results in the specialized literature, this article is likely to advance the state-of-the-art tunable metastructures for vibration mitigation and sound attenuation.

## Figures and Tables

**Table 1:** The temperature-dependent Young’s modulus from the DMA test.

T (°C)	30	40	50	60	70	80	90
E (MPa)	3350	3280	3166	2554	48	18	14

**Table 2:** The thermal expansion coefficient of each layer for different printing speeds.

*α_i_* (1/°C)		*S_p_* (mm/s)		
	10	20	40	70
$\alpha_{1}$	−0.0006	−0.0016	−0.0018	−0.00252
$\alpha_{2}$	−0.0004	−0.0014	−0.0016	−0.00222
$\alpha_{3}$	−0.0002	−0.0011	−0.0013	−0.0022
$\alpha_{4}$	−0.00009	−0.0008	−0.0011	−0.00172
$\alpha_{5}$	−0.00007	−0.0006	−0.0008	−0.00152
$\alpha_{6}$	−0.00005	−0.0004	−0.0005	−0.00122

**Table 3:** The constant of thermal expansion interpolation function for each printing layer.

Layer		Coefficient			
	$C_{1}\;(10^{-8})$	$C_{2}\;(10^{-6})$	$C_{3}(10^{-3})$	$C_{4}\;(10^{-3})$	R−Square
1	−3.1018	4.125	−0.1845	081	0.9722
2	−3.6	4.739	−0.2039	1.16	0.9824
3	−3.44	4.331	−0.1834	1.198	0.9864
4	−2.2	2.917	−0.135	0.9653	0.9917
5	−1.744	2.159	−0.0097	0.6798	0.989
6	−0.804	0.8966	−0.0051	0.2867	0.9716

**Table 4:** The geometric parameters of the beam-like 4D structures after the heating–cooling process.

Method	*S_p_* (mm/s)	$R_{1}(mm)$	$R_{2}(mm)$	$R_{3}(mm)$
**Experiment**	10	29.8	28.2	3.1
	20	29.3	19	8.3
	40	29.1	16.3	9.2
	70	29.0	7.1	10.5
**FE COMSOL Multiphysics**	10	29.7	28.3	3.2
	20	29.4	19.1	8.4
	40	29.2	16.2	9.1
	70	28.9	7.0	10.4
**In-house FE method**	10	29.9	28.3	3.0
	20	29.1	19.2	8.4
	40	29.4	16.3	9.2
	70	29.2	7.2	10.3
